# In Memorium: Swamy Laxminarayan [1939–2005]

**DOI:** 10.1186/1475-925X-4-57

**Published:** 2005-10-05

**Authors:** Kenneth R Foster, Luis G Kun

**Affiliations:** 1 Department of Bioengineering, University of Pennsylvania, 220 South 33rd Street, Philadelphia, PA 19104, USA; 2 IRM College, National Defense University, Fort McNair, Washington, DC 20319, USA

## Abstract

Swamy Narasimha Laxminarayan, known to his many friends and colleagues as Swamy, passed away on September 29, 2005. He was one of the most prominent biomedical engineers on the international scene, and contributed immensely to the globalization of this new field.

Swamy grew up in Bangalore, India. Records list his birthdate as May 24, 1939, although he has reportedly confessed to colleagues that he misrepresented his age to gain early admission to university; his real birthdate may be as late as 1943. He received his undergraduate degree in physics and mathematics from the University of Mysore, India (1957) and Masters (1967) and Ph.D. (1972) from the University of Southampton in the UK. His Ph.D. thesis concerned on-line signals processing and physiological systems, interests that he maintained through his entire professional career.

Beginning in 1970, Swamy held research faculty positions at two universities in the Netherlands (1970–78). A large part of his career was spent at the University of Medicine and Dentistry with an adjunct appointment at the New Jersey Institute of Technology, both in Newark, New Jersey, where he led programs in research computing and health care informatics. He joined Idaho State University in 2002, where he was a Research Associate Professor at the university and chief of Biomedical Information Engineering at Telehealth Idaho. He had numerous other affiliations along the way, including several adjunct clinical appointments, and visiting or honorary appointments at the Technical University of Brno (Czech Republic) and Tsinghua University (China), as well as several affiliations with private industry.

Swamy was a prolific researcher. His resume lists more than 300 technical papers in fields as diverse as biomedical engineering, medical image processing, telehealth, bioterrorism and homeland security. These include numerous original papers, together with a great many conference proceedings and special issues that he edited. Most recently, he was awarded a $3.8 M grant for developing training materials to combat bioterrorism.

Swamy's contributions to engineering are varied and prominent. Many were connected with his enthusiastic leadership in engineering societies and editorial work with professional journals. He was the Founding Editor-in-Chief of the IEEE Transactions on Information Technology in Biomedicine, and served on the editorial boards or as associate editor of numerous other biomedical engineering journals. He had many leadership roles in the Institute of Electronics and Electrical Engineers (IEEE). Most of his work over the years was with the IEEE Engineering in Medicine and Biology Society, but he also held leadership roles in the IEEE Computer and Communications Societies, and in the IEEE at large. He was also active in a host of other societies in medical informatics, telemedicine, medical instrumentation, and related fields, where he worked tirelessly to organize projects related to biomedicine, and to promote individuals' careers and professional recognition.

Swamy's immense contribution came from his ability to move people and projects along, propelled by his incredible enthusiasm and assisted by a vast network of friends around the world. For many years, he was a prominent figure in organizing international meetings in biomedical engineering, and he was exceedingly effective in bringing scientists and engineers from around the world together at these conferences. He was a longtime leader in the International Federation for Medical and Biological Engineering. Biomedical engineering, which began largely as an American enterprise, now has a global scope, thanks in no small part to Swamy's work over the years.

Swamy received numerous awards, including the Purkinje award (Czech Academy of Medical Societies, 1994) for 'pioneering contributions in the field of advanced computer applications to cardiovascular, neuro and pulmonary physiology and for international leadership in information technology in medicine and healthcare', the IEEE 3rd Millennium Medal and career achievement awards, and fellowship in the American Institute of Medical and Biological Engineering. This past spring, Swamy was honored by Idaho State University as a Distinguished Researcher.

A dedicated family man, Swamy is survived by his wife Marijke, daughter Malini, and son, Vinod. He will also be very much missed by his many friends and colleagues throughout the world.

**Figure 1:**
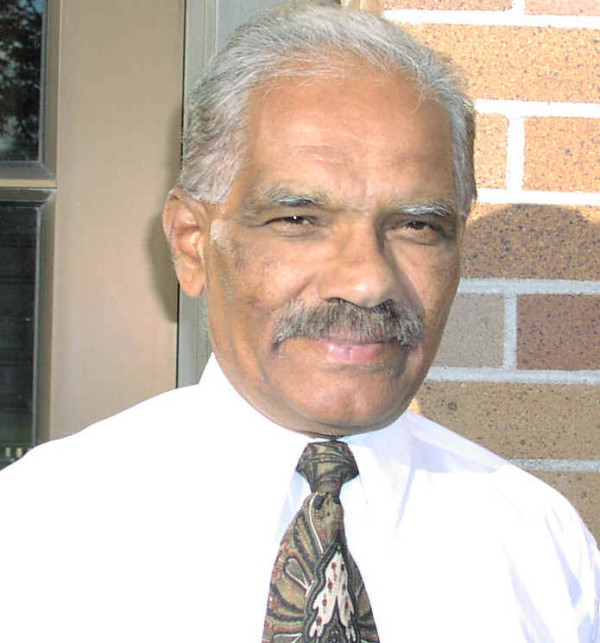
Swamy Laxminarayan [1939–2005]

